# Clinical-Pathological Conference Series from the Medical University of Graz: Case No 160: 33-year-old woman with tetraparesis on Easter Sunday

**DOI:** 10.1007/s00508-016-1085-7

**Published:** 2016-09-28

**Authors:** Elisabeth Fabian, Dietmar Schiller, Andreas Tomaschitz, Cord Langner, Stefan Pilz, Stefan Quasthoff, Reinhard B. Raggam, Rainer Schoefl, Guenter J. Krejs

**Affiliations:** 1 Division of Gastroenterology and Hepatology, Department of Internal Medicine III, Medical University of Vienna, Vienna, Austria; 2 Department of Internal Medicine IV, Elisabethinen Hospital, Linz, Austria; 3 Division of Cardiology, Department of Internal Medicine, Medical University of Graz, Graz, Austria; 4 Department of Pathology, Medical University of Graz, Graz, Austria; 5 Division of Endocrinology and Metabolism, Department of Internal Medicine, Medical University of Graz, Graz, Austria; 6 Department of Neurology, Medical University of Graz, Graz, Austria; 7 Division of Angiology, Department of Internal Medicine, Medical University of Graz, Graz, Austria; 8 Division of Gastroenterology and Hepatology, Department of Internal Medicine, Medical University of Graz, Auenbruggerplatz 15, 8036 Graz, Austria

**Keywords:** Primary aldosteronism, Conn syndrome, Hypokalemic paralysis

## Presentation of case

### Dr. D. Schiller:

During the preceding 4 weeks the patient noticed progressive weakening in all four extremities, first in the upper extremities and the shoulder girdle and then in the lower extremities, causing three falls during the 2 days before admission on Easter Sunday, when tetraparesis was diagnosed in the emergency room. On admission the patient (height 161 cm, weight 71 kg) was alert, oriented and afebrile. Muscle strength was graded 1–2 (grade 0 = total paralysis, grade 5 = normal muscle contraction against full resistance) in all four extremities. Sensory function was normal in all extremities and cranial nerve function was unremarkable. Blood pressure was 180/100 mmHg with a regular pulse of 88 beats per minute. The rest of the physical examination was normal. The patient denied double vision, dysphagia and dysarthria, myalgia, dysuria, dyschezia and recent trauma (except for the three falls shortly before admission), but she had experienced polyuria for the last 2 years. Arterial hypertension had been diagnosed 4 years previously and treated with carvedilol 25 mg and ramipril/hydrochlorothiazide 5/25 mg q.d. Electrocardiography showed sinus rhythm with normal P waves and QRS complexes but clearly flattened T waves in the presence of U waves. Magnetic resonance imaging (MRI) of the brain, the cervical spine and the medulla was normal. Cerebrospinal fluid analysis did not show pleocytosis or increased protein concentration. Further laboratory results: normal red and white blood cell counts except for neutrophilic granulocytosis (16.8 g/l), sodium 144 mmol/l (normal: 132–145 mmol/l), potassium 1.5 mmol/l (normal: 3.5–5.5 mmol/l), creatinine 0.9 mg/dl (normal: 0.5–0.9 mg/dl), aspartate aminotransferase (AST) 311 U/l (normal: 15–37 U/l), alanine aminotransferase (ALT) 121 U/l (normal: 12–34 U/l), creatine kinase (CK) 11,788 U/l (normal: <145 U/l), plasmatic coagulation parameters and markers of inflammation were all within normal limits.

A panel of diagnostic tests was performed.

## Differential diagnosis

### Dr. A. Tomaschitz:

When a patient presents with tetraparesis, the physician might primarily suspect a neurological cause or a neurodegenerative disease, such as neurosarcoidosis. Rheumatologic diseases and infections involving the central nervous system (CNS), e.g. human immunodeficiency virus infection, cytomegalovirus infection or tuberculosis would be further considerations. As, however, there is no evidence that any of these apply to our patient, the outstanding laboratory findings of hypokalemia (1.5 mmol/l, normal: 3.5–5.5 mmol/l) and rhabdomyolysis (CK 11,788 U/l, normal: <145 U/l), should be addressed. Typical endocrine conditions presenting with hypokalemia and rhabdomyolysis resulting in tetraparesis include (1) thyrotoxicosis, i.e. thyrotoxic periodic paralysis, which is a rare clinical manifestation of hyperthyroidism and presents with sudden onset of paralysis associated with severe hypokalemia, (2) Cushing syndrome, i.e. hypokalemia due to increased renal potassium excretion caused by the mineralocorticoid effects of cortisol and (3) massive intake of glucocorticoids, which very rarely results in hypokalemic paralysis.

In this hypertensive patient, hyperaldosteronism (primary aldosteronism) should also be considered. Primary aldosteronism is defined as inappropriately high and (relatively or absolutely) autonomous aldosterone secretion, which is not adequately suppressed by sodium loading [1]. The clinical features of primary aldosteronism are caused by hypersecretion of aldosterone and increased activation of the mineralocorticoid receptor (MR) in the distal nephron. Importantly, the MR is additionally expressed in a variety of epithelial and non-epithelial extrarenal tissues, such as endothelial cells, vascular smooth muscle cells, cardiac myocytes, endothelial progenitor cells and neutrophils [2]. Both (1) the increased aldosterone-MR mediated renal effects (leading to moderate sodium and water retention and excessive potassium excretion) and (2) aldosterone-MR and direct aldosterone mediated (MR-independent and non-genomic) vascular effects (contributing to e.g. vascular stiffening and endothelial dysfunction) result in arterial hypertension and, variably, hypokalemia [3–5]. Only 9–37 % of patients with primary aldosteronism present with hypokalemia, so that it is not essential for diagnosing aldosterone excess [6]. In severe cases of primary aldosteronism, hypokalemia may be expressed as muscle weakness, particularly in the lower extremities, muscle spasms, palpitations and, rarely, hypokalemic paralysis [7], which might indeed be responsible for our patient’s tetraparesis.

Primary aldosteronism is the most common curable form of arterial hypertension and is reported in approximately 5–10 % of hypertensive patients and in approximately 20 % of those with documented treatment-resistant hypertension [8, 9]. Our patient is young and has a medical history of hypertension obviously resistant to antihypertensive drugs, both of which are important diagnostic clues for primary aldosteronism. In primary aldosteronism, younger hypertensive patients often respond poorly to angiotensin-converting enzyme inhibitors, angiotensin receptor blockers, calcium channel blockers and beta-blockers [10]. The use of different drugs, as in the discussed case, suggests primary aldosteronism.

Due to increased renal loss of H^+^ ions, alkalosis is often observed in primary aldosteronism and hypokalemia followed by secondary hyperparathyroidism due to the hypercalciuric effects of aldosterone is also a common feature of the disease. Unfortunately, the protocol does not include any data on the patient’s acid-base balance and serum calcium levels.

Taken together, severe hypokalemia and hypokalemic paralysis in a young patient with a medical history of treatment-resistant hypertension strongly suggest the diagnosis of primary aldosteronism. As the family history was negative for primary aldosteronism in this case, sporadic forms, i. e. idiopathic unilateral or bilateral adrenal hyperplasia and aldosterone-producing adenomas (Conn syndrome), which is the more severe variant of primary aldosteronism, have to be considered. For biochemical confirmation of the clinical suspicion of primary aldosteronism, the aldosterone to renin ratio (ARR) would have to be determined.

## Dr. A. Tomaschitz’s diagnosis

Hypokalemic paralysis due to primary aldosteronism, most likely Conn syndrome.

## Discussion of case

### Dr. D. Schiller:

When this patient arrived in the emergency room with tetraparesis, several differential diagnoses were considered, including polymyositis, acute intermittent porphyria, polyneuropathy due to thiamine deficiency, a space-occupying lesion in the brain, compression of the spinal cord, myasthenia gravis, botulism and trauma. Polymyositis would have been accompanied by characteristic dermatological features but these were not observed. Acute intermittent porphyria can sometimes lead to paralysis but typically presents with abdominal pain, which was not the case. Malnutrition and alcoholism can cause thiamine deficiency subsequently leading to encephalopathy and polyneuropathy. As malnutrition and encephalopathy were not present in our patient, thiamine deficiency seemed unlikely to have caused tetraparesis. MRI showed no cerebral space-occupying lesion or spinal cord compression. Guillain-Barré syndrome could be excluded by normal cerebrospinal fluid findings. All these neurological considerations could be set aside after the serum potassium level of 1.5 mmol/l was reported with some delay, as the laboratory staff had found the initial result implausible and repeated the analysis.

This very low serum potassium level led to the diagnosis of hypokalemic paralysis. Generally, hypokalemia can result from increased renal or gastrointestinal loss or increased cellular uptake (Table 1). Hypokalemia is often associated with QT prolongation and the risk of ventricular tachycardia (particularly torsades de pointes), which can be further aggravated by the administration of drugs known to prolong the QT interval [11]. Hypokalemia in patients with hypertension should initiate a search for primary hyperaldosteronism. After discontinuation of diuretics screening is performed by measuring the plasma levels of aldosterone and renin to calculate the ARR (Table 2). This patient’s ARR was significantly increased (20.4, normal: <12), indicating primary aldosteronism. As the ARR is only a screening tool, the definite diagnosis of primary aldosteronism has to be confirmed by additional diagnostic tests including suppression tests with saline, fludrocortisone and captopril and the oral salt loading test. In the first three tests, post-suppression serum aldosterone concentration is used as a diagnostic criterion; the oral salt loading test assesses 24-h urinary aldosterone excretion [7]. With the saline suppression test, we found a post-suppression plasma aldosterone concentration of 55 ng/dl (cut-off value for aldosterone: >10 ng/dl), which clearly confirmed primary aldosteronism in our patient.

**Table 1:** Major causes of hypokalemia

Increased gastrointestinal loss	Increased urinary loss	Increased entry into cells
Vomiting Diarrhea Tube drainage Laxative abuse	Diuretics Primary mineralocorticoid excess Loss of gastric secretion Non-resorbable anions Renal tubular acidosis Hypomagnesemia Amphotericin B Salt-wasting nephropathies (including Bartter and Gitelman syndromes) Polyuria	Elevation in extracellular pH Increased availability of insulin Elevated beta-adrenergic activity, stress or beta-agonists Hypokalemic periodic paralysis Marked increase in blood cell production Hypothermia Chloroquine intoxication

**Table 2:** Causes of hypokalemia combined with hypertension in relation to aldosterone and renin

Aldosterone ↑ Renin ↓	Aldosterone ↑ Renin ↓	Aldosterone ↑ Renin ↑
Hypercortisolism Congenital adrenal hyperplasia	Primary aldosteronism	Secondary aldosteronism Diuretics Cirrhosis Heart failure Renovascular arterial hypertension Malignant arterial hypertension Renin-secreting tumor

Hypersecretion in primary aldosteronism is often caused by a single aldosterone-producing adenoma in one adrenal gland: MRI revealed a unilateral adenoma, 2 cm in diameter in the left adrenal gland. This prompted the diagnosis of classical solitary Conn adenoma.

Bilateral selective adrenal venous sampling (AVS, i.e. the selective blood sampling from adrenal veins and a peripheral site via a percutaneous femoral vein approach and the comparison of the cortisol-corrected aldosterone secretion between the two sides using the lateralization index) is the gold standard procedure for differentiating unilateral (surgically curable) and bilateral adrenal disease [12]. The AVS is a challenging procedure (particularly the cannulation of the right adrenal vein) and the success rate greatly depends on the expertise of the angiographer. In many centers the right adrenal vein is successfully cannulated in 50–80 % of cases [13, 14]. Only a few centers report success rates >95 % [15]. Unlike the left adrenal vein, which can be easily identified by its drainage into the superior aspect of the left renal vein, the right adrenal vein drains directly into the inferior vena cava in the neighborhood of a number of small vessels (e.g. accessory hepatic, phrenic and retroperitoneal veins) that may look like the right adrenal vein [16]. Moreover, the right adrenal vein is small and short, so that catheters can easily become dislodged during blood collection. In our patient AVS clearly suggested a left unilateral adenoma as indicated by MRI. Within 3 weeks after minimally invasive left-sided adrenalectomy the patient’s blood pressure decreased from 180/100 mmHg to 120/80 mmHg and serum potassium levels returned to normal. In 30–40 % of patients with Conn syndrome adrenalectomy results in permanent normalization of the blood pressure without further need of medication, as was also the case with our patient.

### Dr. C. Langner:

**Fig. 1:**
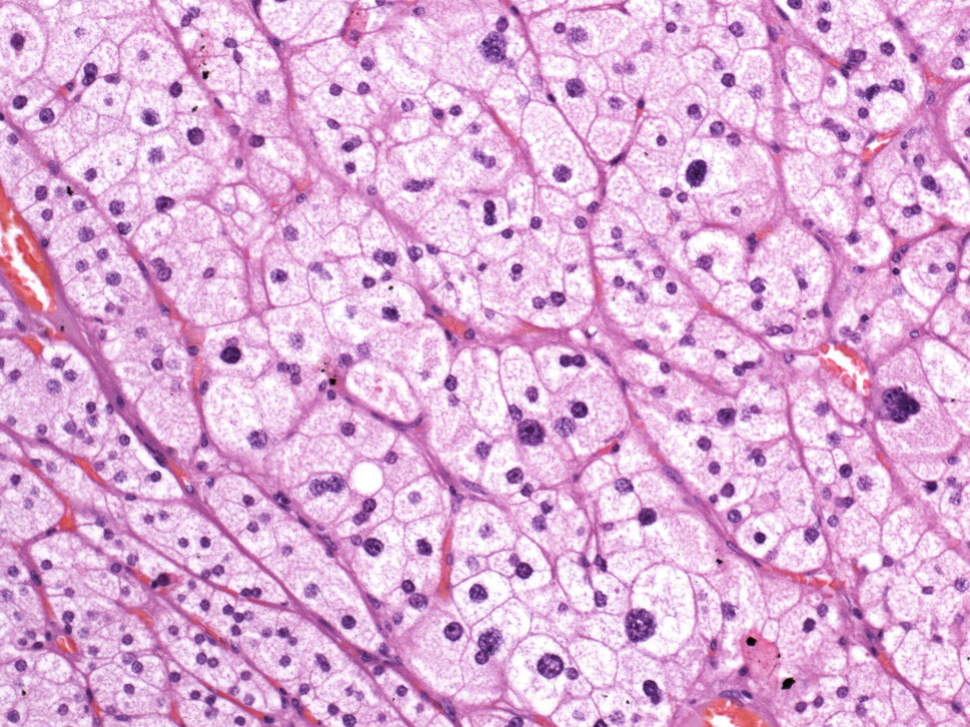
Typical adrenocortical adenoma composed of large cells with abundant foamy cytoplasm and distinct cell borders. Note coarse nuclear chromatin, variation in nuclear size and occasional binucleated cells but no mitotic figures (H&E, ×100)

Histologically, the adrenal gland can be divided into the medulla, which shows a relatively homogeneous structure and the cortex with three concentric zones (zonae glomerulosa, fasciculata and reticularis). Due to their different embryologic origins, the cortex and medulla produce different hormones. While catecholamines are synthesized in the medulla, steroid hormones, such as mineralocorticoids, glucocorticoids and sex steroids are synthesized in the zonae glomerulosa, fasciculata and reticularis of the cortex, respectively, with an overlap in hormones synthesized by the zonae fasciculata and reticularis. Our patient’s adenoma showed enlarged cells with pale cytoplasm and centrally placed nuclei with marked variation in size and there were also binucleated cells but no mitotic figures (Fig. 1).

### Dr. G.J. Krejs:

In this case tetraparesis caused by severe hypokalemia was the outstanding clinical feature. Dr. Quasthoff will discuss this from the neurological point of view.

### Dr. S. Quasthoff:

In patients with periodic paralysis the medical history is essential for neurologists. Exclusion of diagnoses, such as a cerebral space-occupying lesion, compression of the spinal cord and spinal cord diseases, neuromuscular diseases, such as myasthenia gravis, botulism, periodic paralysis due to hyperthyroidism (i.e. thyrotoxicosis) and those discussed earlier will limit the correct neurological diagnosis in a patient with tetraparesis to psychogenic or hypokalemic paralysis. With an annual incidence of 1 in 1 million inhabitants, hypokalemic tetraparesis is seen only very rarely. Hypokalemia has different underlying causes, which have to be distinguished (Table 1). Patients with hypokalemic periodic paralysis may experience paralytic episodes with concomitant hypokalemia (<2.5 mmol/l) and may occasionally develop late-onset proximal myopathy. The paralytic attacks are characterized by reversible flaccid paralysis usually leading to paraparesis or tetraparesis but sparing the respiratory muscles and the heart [17].

The symptoms in hypokalemic paralysis can last from minutes to hours or several days. Some individuals have only one episode in their lifetime but more commonly, crises occur repeatedly: daily, weekly, monthly or even less often. Events that are associated with an increased release of epinephrine or insulin can precipitate hypokalemic paralysis as they cause increased movement of potassium into cells and a subsequent decrease in serum potassium [18, 19]. Major triggering factors are either rest after strenuous physical activity, stress or a high carbohydrate load. From my personal experience I remember two cases of hypokalemic paralysis that were both associated with these typical triggering factors. The first patient was a student on a summer internship involving heavy manual labor; his daily diet was very high in carbohydrates including up to 3 l of sugar-sweetened beverages. This constellation finally led to hypokalemic periodic paralysis. The second case was a bride who presented with hypokalemic paralysis on her wedding day. Again, stress, physical activity (in this case dancing) and a high-carbohydrate meal were the triggering factors. It is of interest that our patient developed paralysis on Easter Sunday, when people typically have a large festive meal.

When hypokalemic periodic paralysis is suspected, the diagnosis has to be confirmed by electrophysiology, provocation tests and/or genetic analysis. Detailed information on this testing is available elsewhere [20]. The exercise test determines muscle strength by electromyography after nerve stimulation. The characteristic changes are important findings for defining the cause of paralysis, i.e. which ion channel is affected. Alternatively, a glucose provocation test, which aims to decrease serum potassium levels to below 3 mmol/l by oral administration of 2 g glucose/kg body weight and 10–20 IU insulin subcutaneously or i.v. administration of 1.5–3 g glucose/kg body weight and insulin over a period of 60 min, can confirm the diagnosis of hypokalemic periodic paralysis. In hypokalemic paralysis, serum concentrations of potassium are typically low (0.9–2.5 mmol/l) during attacks but not in between. Moreover, a positive family history is consistent with autosomal dominant inheritance. Of all individuals meeting the diagnostic criteria for hypokalemic periodic paralysis, approximately 60 % have mutations in the calcium channel *CACNA1S* gene, 20 % in the sodium channel *SCN4A *gene and 3.5 % in the potassium *KCNJ18* gene [17]. Depending on the mutation, paralysis in the affected patients may be due to hyperkalemia or hypokalemia (Table 3). It is important to know that in patients with hypokalemic periodic paralysis, CK levels may be increased even without myotonia, as in our patient.

**Table 3:** Clinical features of hypokalemic and hyperkalemic periodic paralysis

Hypokalemic periodic paralysis	Hyperkalemic periodic paralysis
Usually presents in adolescence Attacks can be prolonged, usually occurring on awakening and accompanied by hyporeflexia Potassium is low during attacks Myotonia is never present Possible proximal myopathy Rarely associated mild sensory axonal polyneuropathy *Electrophysiology:* Rarely associated mild sensory axonal polyneuropathy Never myotonia Effects of cooling unknown Short exercise test causes no decrement Long exercise test produces immediate increase in CMAP, especially if initial CMAP is low, followed by progressive decline in CMAP by 50 % over 20–40 min with most of the decline in the first 20 min RNS causes no decrement unless after prolonged exercise	Attacks of periodic weakness provoked by fasting, rest after exercise or cold Attacks are brief, lasting minutes to hours and accompanied by hyporeflexia Potassium is usually elevated during attacks Attack relieved by ingesting carbohydrates Development of progressive weakness during adulthood *Electrophysiology:* May not have myotonia between attacks Rarely myopathic units Myotonia seen early in the attack but disappears as weakness progresses Muscle cooling has no effect Short exercise test produces no decrement Long exercise test produces immediate increase in CMAP, especially if initial CMAP is low, followed by progressive decline in CMAP by 50 % over 20–40 min with most of the decline in the first 20 min RNS causes no decrement unless after prolonged exercise

*CMAP* compound muscle action potential, *RNS* repetitive nerve stimulation

Mutations in the potassium channel *KCNJ2* gene (inward-rectifier potassium ion channel) often presents clinically as Andersen-Tawil syndrome; however, penetrance is extremely variable, with some carriers of the mutation displaying little or no phenotypic expression [21]. This rare syndrome is characterized by a triad of episodic flaccid muscle weakness (periodic paralysis), ventricular arrhythmia with prolonged QT interval and skeletal anomalies [22]. Neurological presentation commonly includes episodic weakness of skeletal muscles in a generalized pattern with sparing of bulbar and respiratory musculature and reflexes may be absent or diminished during the episodes of weakness. Electrophysiological evaluation of the nerves is of great diagnostic value, as abnormalities are seen with sensitive testing in about 80 % of cases. Classical electrocardiographic abnormalities include prominent Q waves, prolonged QT and QU intervals, ventricular arrhythmias, such as premature ventricular contractions, polymorphic ventricular tachycardia and bidirectional ventricular tachycardia. Skeletal anomalies in the syndrome are micrognathia, retrognathia, clinodactyly, syndactyly, low-set ears and hypertelorism [23].

Table 4 provides an overview of hypokalemic and hyperkalemic causes of paralysis, corresponding characteristic symptoms, affected ion channels and treatment options.

**Table 4:** Overview of hypokalemic and hyperkalemic paralysis, characteristic symptoms, affected ion channels and treatment options

	Hypokalemic periodic paralysis	Thyrotoxic hypokalemic periodic paralysis	Hyperkalemic periodic paralysis	Potassium-sensitive myotonia	Andersen-Tawil syndrome
*Channel or gene defect*	Ca^++^: CACNA1S α_1_-subunit (or rare: Na^+^: SCN4A; K^+^: KCNE3, KCNJ2, KNCJ5)	K^+^: KCNJ18 (Kir2.6)	Na^+^: SCN4A α-subunit (or ﻿K^+^: KCNE3)	Na^+^: SCN4A α-subunit	K^+^: KCNJ2 (Kir2.1)
*Inheritance/locus*	Dominant 1q32.1	Dominant 17q11.2	Dominant 17q23.3	Dominant 17q23.3	Dominant 17q23.3
*Functional* *defect*	?	?	Reduced fast and slow inactivation	Mild reduction of fast inactivation	↓ K^+^ conduction
*Penetrance*	↓ Females	↓ Females	High	High	Variable
*Onset*	5–35 years	20–40 years	<10 years	<10 years	2–18 years
*Weakness duration*	2–24 h	Hours to days	30 min–4 h	None	1–36 h
*Maximum weakness*	Severe	Mild to severe	Mild to severe	None	Moderate
*Cold*	± ↑ Paralysis	± ↑ Paralysis	± ↑ Paralysis	↑ Paralysis	?
*K* ^+^ *-effects*	↓ Paralysis	↓ Paralysis	↑ Paralysis	↑ Paralysis	↑ Paralysis
*Attack precipitants*	Carbohydrates, rest after activity	Carbohydrates, rest after activity	Fasting	↑ K^+^	K^+^
*Myotonia*	Rarely eyelids only	None	± Present Exercise: ↓	Mild to severe Exercise: ↑	None
*Large muscles*	No	No	Yes	Yes	No
*Sensory symptoms*	Absent	Absent	Present	?	Absent
*Drug treatment*	K^+^ Acetazolamide Dichlorphenamide	Therapy of thyrotoxicosis Beta-adrenergic blockade	Thiazide Acetazolamide Dichlorphenamide	Mexiletine Thiazide	?

### Dr. G.J. Krejs:

Hypokalemia is an important diagnostic laboratory finding in several diseases and responsible for specific clinical features. But how often do we actually see hypokalemia in our clinical routine? Dr. Raggam will present the experience with potassium measurements and the frequency of hypokalemia at the University Medical Center in Graz.

### Dr. R.B. Raggam:

From January to November 2015 a total of 349,418 serum potassium analyses were performed at the University Medical Center in Graz. Normal potassium levels range from 3.5 to 5.5 mmol/l; in 0.15 % (*n* = 531) of samples analyzed, the potassium concentration was between 2.1 and 2.5 mmol/l and only 0.02 % (*n* = 85) had a concentration ≤2.0 mmol/l. With 1.5 and 1.7 mmol/l, the lowest levels were found in psychiatric patients. Most frequently levels ≤2.0 mmol/l were seen in patients in intensive care units and on medical and surgical wards.

For biochemical screening for primary aldosteronism in at-risk populations, there are no internationally accepted standardized assays that are calibrated against a standard reference substance. Calculation of the ARR is, however, a widely accepted approach [24]. The ratio has superior test characteristics to either plasma aldosterone concentration or plasma renin concentration (or activity) alone [25] and it shows less intra-day and inter-day variation. It is also less influenced by preanalytical variables, such as salt intake, diuretic exposure and posture prior to collection [26].

Calculated ARRs from different laboratories are, however, hardly comparable because the results are significantly influenced by (1) the assays used to determine aldosterone and renin and (2) the prevailing preconditions for blood sampling. The ARR can be calculated from both the direct “active” renin concentration (aldosterone to direct active renin concentration ratio, AARR) and the plasma renin activity (aldosterone to renin activity ratio, ARAR). It is important to know that both parameters are different metrics of the renin-angiotensin-aldosterone system and will therefore show different positive screening rates [27]. As direct active renin concentrations are biased by various factors [7], some experts tend to use plasma renin activity rather than renin concentration to calculate the ARR [28, 29], even though screening using the renin concentration can also be effective [30]. The diagnostic accuracy of the ARAR is, however, limited by conditions that affect both renin concentrations (e.g. negative feedback inhibition by angiotensin II) and angiotensinogen concentrations (increased during pregnancy, glucocorticoid excess and estrogen administration; decreased in liver disease) [31]. In contrast, the advantages of ARAR over AARR may be a better low-end precision and a more complete assessment of the renin-angiotensin-aldosterone system tone [7]. Glinicki et al. recently suggested a similar diagnostic accuracy of the ARAR and AARR [32]. In summary, for practical and economic reasons ARAR can be replaced by the aldosterone to direct active renin concentration ratio (AARR). Due to these analytical complexities, the ARR should always be interpreted in the context of the full clinical picture including medical and family history of early cardiovascular events, of early onset of arterial hypertension, severity of hypertension, resistance to antihypertensive drugs, presence of adrenal adenoma, absolute aldosterone concentration, the presence of electrolyte abnormalities and responsiveness to aldosterone antagonists [7]. When screening is positive, a confirmatory aldosterone suppression test must be performed to definitely diagnose primary aldosteronism.

### Dr. G.J. Krejs:

At the University Medical Center of Graz only 1 out of approximately 350,000 measurements per year yields a serum potassium level as low as 1.5 mmol/l as observed in our patient. In the past 45 years of my medical career I have just once seen a patient with a potassium level as low as 1.5 mmol/l. That patient suffered from Verner-Morrison syndrome, i.e. watery diarrhea hypokalemia hypochlorhydria (WDHH) syndrome, also known as VIPoma (vasoactive intestinal peptide) or pancreatic cholera [33] and experienced hypokalemic paralysis caused by massive gastrointestinal potassium loss. He was paralyzed to such a degree that he could not lift the telephone receiver to call for help.

The prevalence of hypertension has increased over the past decades and currently affects about 1 billion people worldwide [34]. Although most cases of hypertension are “essential” or “idiopathic,” some cases have an identifiable cause, representing secondary hypertension for which curative therapy can be offered. The most common form of secondary hypertension is primary aldosteronism [5]. Primary aldosteronism was first described in 1955 by Conn at Yale University, who reported a 34-year-old woman with hypertension, intermittent paralysis, hypokalemia and metabolic alkalosis [35]. The patient further showed increased activity of urinary salt-retaining corticoid and was cured by removal of a benign adrenal adenoma [36]. Initially Conn suggested that about 20 % of hypertensive patients might be affected by primary aldosteronism but later revised his estimate to 10 % [37]. Currently it is suggested that in 5–13 % of patients with hypertension the cause is primary aldosteronism with higher rates in younger hypertensive patients [5, 7]. Dr. Pilz is an endocrinologist and will present some important facts on primary aldosteronism and tell us about experiences with this disease at the University Medical Center of Graz.

### Dr. S. Pilz:

Primary aldosteronism is characterized by aldosterone concentrations that are inappropriately high in relation to renin and that are not adequately suppressible by sodium loading, which would normally reduce aldosterone secretion [7]. Identification of primary aldosteronism and its specific forms (Table 5) is essential because affected patients have higher cardiovascular morbidity and mortality than age, sex and blood pressure-matched patients with essential hypertension [10, 42]. High aldosterone levels have adverse cardiovascular effects that are independent of hypertension and include endothelial dysfunction, left ventricular hypertrophy and fibrosis of the kidney, heart and blood vessels [43]. Early detection of primary aldosteronism by screening of hypertensive patients is important because it will allow targeted and effective treatment and will so reduce the excess cardiovascular risk [44, 45].

**Table 5:** Subtypes of primary aldosteronism and their frequency [5, 7, 38]

Subtype	Relative frequency (%)
*Sporadic forms*	
Idiopathic adrenal hyperplasia	60–65
Aldosterone-producing adenoma (Conn syndrome)	30
Primary unilateral adrenal hyperplasia	2–3
Aldosterone-producing adrenocortical carcinoma	1
Aldosterone-producing ovarian tumor	<1
*Familial forms*	
Familial hyperaldosteronism type I (glucocorticoid-remediable aldosteronism) caused by a fusion gene between *CYP11B2* (aldosterone synthase) and *CYP11B1* (11-ß-hydroxylase) resulting in excessive aldosterone production in response to adrenocorticotropin [39]	<1
Familial hyperaldosteronism type II (familial occurrence of aldosterone-producing adenoma or bilateral idiopathic hyperplasia or both; genetic cause is under investigation)	Unknown
Familial hyperaldosteronism type III (non-glucocorticoid-remediable hyperaldosteronism) caused by mutations of the inward rectifier potassium channel 4 (GIRK4) which is encoded by the KCNJ5 gene [40, 41]	Unknown
Ectopic aldosterone-producing adenoma or carcinoma	<0.1

According to the Endocrine Society clinical practice guidelines, testing for primary aldosteronism should be considered in any of the following circumstances: (1) sustained blood pressure above 150/100 mmHg on each of three measurements obtained on different days, (2) hypertension (blood pressure >140/90 mmHg) resistant to three conventional antihypertensive drugs (including a diuretic), (3) controlled blood pressure (<140/90 mmHg) on four or more antihypertensive drugs, (4) arterial hypertension and spontaneous or diuretic-induced hypokalemia, (5) hypertension and adrenal incidentaloma, (6) hypertension and sleep apnea, (7) hypertension and a family history of early onset hypertension or cerebrovascular accident at a young age (<40 years) and (8) all hypertensive first-degree relatives of patients with primary aldosteronism [12]. As already described by Drs. Schiller and Raggam, primary aldosteronism is usually diagnosed by measuring plasma aldosterone and direct active renin concentrations or renin activity and calculating the ARAR or AARR. If ARAR or AARR is elevated, confirmatory aldosterone suppression should usually be conducted to verify autonomous aldosterone production and thus confirm the diagnosis of primary aldosteronism. When testing for primary aldosteronism it is recommended to withdraw mineralocorticoid receptor antagonists (e.g. spironolactone and eplerenone) and amiloride and triamterene for at least 4 weeks before blood sampling to avoid spurious results. Potassium should also be normalized before testing because hypokalemia strongly suppresses aldosterone secretion. If a young patient (<35 years) has marked aldosterone excess and spontaneous hypokalemia along with plasma renin levels below detection levels there may be no need for further confirmation testing [5]. Beyond these diagnostic procedures for primary aldosteronism, it should be emphasized that one important differential diagnosis for patients with profound hypokalemia and hypertension is ectopic, i.e. adrenocorticotropic hormone dependent Cushing syndrome that is usually accompanied by significant metabolic alkalosis and very rapid disease progression requiring early diagnosis and treatment [46]. Some drugs such as beta-adrenergic blockers, angiotensin-converting enzyme inhibitors or angiotensin receptor blockers may also impact on the ARR and it is desirable to withdraw these drugs for at least 4 weeks before testing.

In addition to renin (activity) and aldosterone levels for calculation of ARR or AARR, analysis of parathyroid hormone may be of additional diagnostic value in primary aldosteronism. Patients with significant primary aldosteronism frequently present with elevated parathyroid hormone levels that may be due to increased renal and fecal calcium loss, metabolic alkalosis or direct effects on the parathyroid glands mediated by aldosterone [47].

The overall prevalence of primary aldosteronism, which of course greatly depends on the diagnostic criteria applied (e.g. ARR cut-offs), is relatively low at our center; according to the Graz endocrine causes of hypertension (GECOH) study approximately 3–5 % of patients referred for screening for endocrine hypertension are diagnosed with primary aldosteronism per year at the University Medical Center in Graz [48, 49]*. *In absolute numbers this means 5 patients are diagnosed here per year; however, appropriate and targeted screening of suspicious patients is pivotal for early diagnosis and adequate therapy.

### Dr. G.J. Krejs:

In summary, this interesting case showed that primary aldosteronism due to an aldosterone-producing adenoma (Conn syndrome) led to severe hypokalemic paralysis. Primary aldosteronism is not often seen in the daily routine but is detected in about five patients per year at our medical center and hypokalemic paralysis is seen even less often. Early identification of patients with primary aldosteronism and the correct subtype is important for the choice of the best therapy. In the discussed patient minimally invasive adrenalectomy was curative.

### Dr. A. Tomaschitz:

As a final comment on this case: hypokalemic tetraparesis in primary aldosteronism is rare but physicians should nevertheless be aware of this clinical feature. Regarding primary aldosteronism it is important to know that hypokalemia is only found in 37 % of patients with primary aldosteronism and that hypokalemia is more common when the disease has progressed [6, 50]. As potassium levels are often within the normal range, hypokalemia is an insensitive diagnostic tool for primary aldosteronism. The ARR or AARR are the acceptable approaches for detecting primary aldosteronism but a confirmatory aldosterone suppression test must be performed before treatment can be initiated.

## Final diagnosis

Hypokalemic paralysis due to primary aldosteronism (Conn syndrome).

## References

[1] Funder JW, Carey RM, Fardella C, Gomez-Sanchez CE, Mantero F, Stowasser M, Young WF, Montori VM, Endocrine Society (2008). Case detection, diagnosis, and treatment of patients with primary aldosteronism: an endocrine society clinical practice guideline. J Clin Endocrinol Metab.

[2] Chao CT, Wu VC, Kuo CC, Lin YH, Chang CC, Chueh SJ, Wu KD, Pimenta E, Stowasser M (2013). Diagnosis and management of primary aldosteronism: an updated review. Ann Med.

[3] Tomaschitz A, Pilz S, Ritz E, Obermayer-Pietsch B, Pieber TR (2010). Aldosterone and arterial hypertension. Nat Rev Endocrinol.

[4] Gordon RD (1994). Mineralocorticoid hypertension. Lancet.

[5] Mattsson C, Young WF (2006). Primary aldosteronism: diagnostic and treatment strategies. Nat Clin Pract Nephrol.

[6] Mulatero P, Stowasser M, Loh K-C, Fardella CE, Gordon RD, Mosso L (2004). Increased diagnosis of primary aldosteronism, including surgically correctable forms, in centers from five continents. J Clin Endocrinol Metab.

[7] Rehan M, Raizman JE, Cavalier E, Don-Wauchope AC, Holmes DT (2015). Laboratory challenges in primary aldosteronism screening and diagnosis. Clin Biochem.

[8] Rossi GP (2011). A comprehensive review of the clinical aspects of primary aldosteronism. Nat Rev Endocrinol.

[9] Calhoun DA, Nishizaka MK, Zaman MA, Thakkar RB, Weissmann P (2002). Hyperaldosteronism among black and white subjects with resistant hypertension. Hypertension.

[10] Milliez P, Girerd X, Plouin PF, Blacher J, Safar ME, Mourad JJ (2005). Evidence for an increased rate of cardiovascular events in patients with primary aldosteronism. J Am Coll Cardiol.

[11] Yap YG, Camm AJ (2003). Drug induced QT prolongation and torsades de pointes. Heart.

[12] Funder J, Carey RM, Mantero F, Murad H, Reincke M, Shibata H, Stowasser M, Young WF (2016). The management of primary aldosteronism: case detection, diagnosis, and treatment: an endocrine society clinical practice guideline. J Clin Endocrinol Metab.

[13] Vonend O, Ockenfels N, Gao X, Allolio B, Lang K, Mai K, Quack I, Saleh A, Degenhart C, Seufert J, Seiler L, Beuschlein F, Quinkler M, Podrabsky P, Bidlingmaier M, Lorenz R, Reincke M, Rump LC, German Conn’s Registry (2011). Adrenal venous sampling: evaluation of the German Conn’s registry. Hypertension.

[14] Harvey A, Kline G, Pasieka JL (2006). Adrenal venous sampling in primary hyperaldosteronism: comparison of radiographic with biochemical success and the clinical decision-making with “less than ideal” testing. Surgery.

[15] Young WF, Stanson AW, Thompson GB, Grant CS, Farly DR, van Heerden JA (2004). Role for adrenal venous sampling in primary aldosteronism. Surgery.

[16] Daunt N (2005). Adrenal vein sampling: how to make it quick, easy, and successful. Radiographics.

[17] Vicart S, Sternberg D, Arzel-Hézode M, Franques J, Bendahhou S, Lory P, Hainque B, Fournier E, Nicole S, Fontaine B (2014). Hypokalemic periodic paralysis. GeneReviews.

[18] Jurkat-Rott K, Lerche H, Lehmann-Horn F (2002). Skeletal muscle channelopathies. J Neurol.

[19] Lin SH, Lin YF, Halperin ML (2001). Hypokalaemia and paralysis. QJM.

[20] Alkaabi JM, Mushtaq A, Al-Maskari FN, Moussa NA, Gariballa S (2010). Hypokalemic periodic paralysis: a case series, review of the literature and update of management. Eur J Emerg Med.

[21] Lange PS, Er F, Gassanov N, Hoppe UC (2003). Andersen mutations of KCNJ2 suppress the native inward rectifier current IK1 in a dominant-negative fashion. Cardiovasc Res.

[22] Tawil R, Ptacek LJ, Pavlakis SG, DeVivo DC, Penn AS, Ozdemir C (1994). Andersen’s syndrome: Potassium-sensitive periodic paralysis, ventricular ectopy, and dysmorphic features. Ann Neurol.

[23] Babu SS, Nigam GB, Peter CS, Peter CS (2015). Andersen-Tawil syndrome: A review of literature. Neurol India.

[24] Tomaschitz A, Pilz S (2010). Aldosterone to renin ratio-a reliable screening tool for primary aldosteronism?. Horm Metab Res.

[25] Tiu SC, Choi CH, Shek CC, Ng YW, Chan FK, Ng CM, Kong AP (2005). The use of aldosterone-renin ratio as a diagnostic test for primary hyperaldosteronism and its test characteristics under different conditions of blood sampling. J Clin Endocrinol Metab.

[26] McKenna TJ, Sequeira SJ, Heffernan A, Chambers J, Cunningham S (1991). Diagnosis under random conditions of all disorders of the renin-angiotensin-aldosterone axis, including primary hyperaldosteronism. J Clin Endocrinol Metab.

[27] Lonati C, Bassani N, Gritti A, Biganzoli E, Morganti A (2014). Measurement of plasma renin concentration instead of plasma renin activity decreases the positive aldosterone-to-renin ratio tests in treated patients with essential hypertension. J Hypertens.

[28] Sealey JE, Gordon RD, Mantero F (2005). Plasma renin and aldosterone measurements in low renin hypertensive states. Trends Endocrinol Metab.

[29] Stowasser M, Gordon RD (2014). The aldosterone-renin ratio: role and problems. Prim Aldosteronism.

[30] Rossi GP, Barisa M, Belfiore A, Desideri G, Ferri C, Letizia C, Maccario M, Morganti A, Palumbo G, Patalano A, Roman E, Seccia TM, Pessina AC, Mantero F, PAPY Study Investigators (2010). The aldosterone-renin ratio based on the plasma renin activity and the direct renin assay for diagnosing aldosterone-producing adenoma. J Hypertens.

[31] Campbell DJ, Nussberger J, Stowasser M, Danser AH, Morganti A, Frandsen E, Ménard J (2009). Activity assays and immunoassays for plasma renin and prorenin: information provided and precautions necessary for accurate measurement. Clin Chem.

[32] Glinicki P, Jeske W, Bednarek-Papierska L, Kruszyńska A, Gietka-Czernel M, Rosłonowska E, Słowińska-Srzednicka J, Kasperlik-Załuska A, Zgliczyński W (2015). The ratios of aldosterone/plasma renin activity (ARR) versus aldosterone/direct renin concentration (ADRR). J Renin Angiotensin Aldosterone Syst.

[33] Fabian E, Kump P, Krejs GJ (2012). Diarrhea caused by circulating agents. Gastroenterol Clin North Am.

[34] Kearney PM, Whelton M, Reynolds K, Muntner P, Whelton PK, He J (2005). Global burden of hypertension: analysis of worldwide data. Lancet.

[35] Conn JW (1955). Presidential address. I. Painting background. II. Primary aldosteronism, a new clinical syndrome. J Lab Clin Med.

[36] Conn JW, Conn ES (1961). Primary aldosteronism versus hypertensive disease with secondary aldosteronism. Recent Prog Horm Res.

[37] Conn JW (1967). The evolution of primary aldosteronism: 1954–1967. Harvey Lect.

[38] Young WF (2003). Primary aldosteronism – treatment options. Growth Horm IGF Res.

[39] McMahon GT, Dluhy RG (2004). Glucocorticoid-remediable aldosteronism. Arq Bras Endocrinol Metabol.

[40] Choi M, Scholl UI, Yue P, Björklund P, Zhao B, Nelson-Williams C, Ji W, Cho Y, Patel A, Men CJ, Lolis E, Wisgerhof MV, Geller DS, Mane S, Hellman P, Westin G, Åkerström G, Wang W, Carling T, Lifton RP (2011). K+ channel mutations in adrenal aldosterone-producing adenomas and hereditary hypertension. Science.

[41] Funder JW (2012). The genetic basis of primary aldosteronism. Curr Hypertens Rep.

[42] Stowasser M, Sharman J, Leano R, Gordon RD, Ward G, Cowely D, Marwick TH (2005). Evidence for abnormal left ventricular structure and function in normotensive individuals with familial hyperaldosteronism type I. J Clin Endocrinol Metab.

[43] Brown NJ (2005). Aldosterone and end-organ damage. Curr Opin Nephrol Hypertens.

[44] Catena C, Colussi G, Nadalini E, Chiuch A, Baroselli S, Lapenna R, Sechi LA (2008). Cardiovascular outcomes in patients with primary aldosteronism after treatment. Arch Intern Med.

[45] Strauch B, Perak O, Zelinka T, Wichterle D, Holaj R, Kasalicky M, Safarik L, Rosa J, Widimsky J (2008). Adrenalectomy improves arterial stiffness in primary aldosteronism. Am J Hypertens.

[46] Schwetz V, Aberer F, Stiegler C, Pieber TR, Obermayer-Pietsch B, Pilz S (2015). Fluconazole and acetazolamide in the treatment of ectopic Cushing’s syndrome with severe metabolic alkalosis. Endocrinol Diabetes Metab Case Rep.

[47] Pilz S, Kienreich K, Drechsler C, Ritz E, Fahrleitner-Pammer A, Gaksch M, Meinitzer A, März W, Pieber TR, Tomaschitz A (2012). Hyperparathyroidism in patients with primary aldosteronism: cross-sectional and interventional data from the GECOH study. J Clin Endocrinol Metab.

[48] Pilz S, Tomaschitz A, Stepan V, Obermayer-Pietsch B, Fahrleitner-Pammer A, Schweighofer N, Portugaller HR, Sourij H, Dobnig H, Meinitzer A, Pieber TR (2009). Graz Endocrine Causes of Hypertension (GECOH) study: a diagnostic accuracy study of aldosterone to active renin ratio in screening for primary aldosteronism. BMC Endocr Disord.

[49] Pilz S, Kienreich K, Gaksch M, Grübler M, Verheyen N, Bersuch LA, Schmid J, Drechsler C, Ritz E, Moosbrugger A, Stepan V, Pieber TR, Meinitzer A, März W, Tomaschitz A (2014). Aldosterone to active Renin ratio as screening test for primary aldosteronism: reproducibility and influence of orthostasis and salt loading. Horm Metab Res.

[50] Rossi GP, Bernini G, Caliumi C, Desideri G, Fabris B, Ferri C (2006). A prospective study of the prevalence of primary aldosteronism in 1,125 hypertensive patients. J Am Coll Cardiol.

